# Utilizing Text Mining, Data Linkage and Deep Learning in Police and Health Records to Predict Future Offenses in Family and Domestic Violence

**DOI:** 10.3389/fdgth.2021.602683

**Published:** 2021-02-17

**Authors:** George Karystianis, Rina Carines Cabral, Soyeon Caren Han, Josiah Poon, Tony Butler

**Affiliations:** ^1^School of Population Health, University of New South Wales, Sydney, NSW, Australia; ^2^School of Computer Science, University of Sydney, Sydney, NSW, Australia

**Keywords:** family and domestic violence, deep learning, big data, text mining, health records, predictive analytics, data linkage

## Abstract

Family and Domestic violence (FDV) is a global problem with significant social, economic, and health consequences for victims including increased health care costs, mental trauma, and social stigmatization. In Australia, the estimated annual cost of FDV is $22 billion, with one woman being murdered by a current or former partner every week. Despite this, tools that can predict future FDV based on the features of the person of interest (POI) and victim are lacking. The New South Wales Police Force attends thousands of FDV events each year and records details as fixed fields (e.g., demographic information for individuals involved in the event) and as text narratives which describe abuse types, victim injuries, threats, including the mental health status for POIs and victims. This information within the narratives is mostly untapped for research and reporting purposes. After applying a text mining methodology to extract information from 492,393 FDV event narratives (abuse types, victim injuries, mental illness mentions), we linked these characteristics with the respective fixed fields and with actual mental health diagnoses obtained from the NSW Ministry of Health for the same cohort to form a comprehensive FDV dataset. These data were input into five deep learning models (MLP, LSTM, Bi-LSTM, Bi-GRU, BERT) to predict three FDV offense types (“hands-on,” “hands-off,” “Apprehended Domestic Violence Order (ADVO) breach”). The transformer model with BERT embeddings returned the best performance (69.00% accuracy; 66.76% ROC) for “ADVO breach” in a multilabel classification setup while the binary classification setup generated similar results. “Hands-off” offenses proved the hardest offense type to predict (60.72% accuracy; 57.86% ROC using BERT) but showed potential to improve with fine-tuning of binary classification setups. “Hands-on” offenses benefitted least from the contextual information gained through BERT embeddings in which MLP with categorical embeddings outperformed it in three out of four metrics (65.95% accuracy; 78.03% F1-score; 70.00% precision). The encouraging results indicate that future FDV offenses can be predicted using deep learning on a large corpus of police and health data. Incorporating additional data sources will likely increase the performance which can assist those working on FDV and law enforcement to improve outcomes and better manage FDV events.

## Introduction

Family and domestic violence (FDV) is a worldwide problem with most FDV perpetrated by men against women resulting in a significant economic and health burden on the community. Estimates suggest that 35% of women worldwide have experienced either physical and/or sexual intimate partner violence in their lifetime (1). In Australia, FDV is the leading cause of morbidity and mortality for women surpassing known risk factors like obesity and smoking (2). In 2018, one in six women and one in 16 men experienced physical and/or sexual violence by their current/former partner and on average, one woman per week is murdered by a current/former partner (3). Research also shows that children exposed to FDV experience long-term effects on their development with an increased risk of mental health issues, learning difficulties and behavioral problems (4). FDV has been linked to high mortality rates, a wide variety of physical injuries ranging from minor traumas to serious injury resulting in hospitalizations (5), and short and long-term mental health consequences including depression, substance abuse, suicide and acts of self-harm (6–8).

The New South Wales Police Force (NSWPF) attends and subsequently records details on thousands of FDV events each year (123,330 in 2017) in their COPS database, an interface for the Computerized Operational Policing System (COPS) that captures and analyses crime information on an organization-wide basis (Chief Inspector Matthew McCarthy, NSWPF, personal communication, June 2018). An FDV event is defined as an incidence of domestic dispute that involves any form of violence or abuse between a person of interest (POI)—an individual accused of perpetrating any form of violence or abuse toward another person—and a victim. Information related to the FDV events is recorded as both a) structured data (fixed fields) that cover demographic information (e.g., name, date of birth, Aboriginal status) for the POI and victim involved in an FDV event, and b) “event narratives” which contain a wealth of features (e.g., abuse types perpetrated by POIs, victim sustained injuries, mental health status, threats) in the form of unstructured text. The text narratives can contain misspellings typographical errors, informal acronyms, jargon, and abbreviations, which can bear ambiguous meanings depending on the context. Although they are used as an aide-memoire for police officers and lawyers should the case proceed to court, to date they rarely have been utilized for research purposes. The information contained within the event narratives can be used to potentially identify trends over time in FDV events and assist in shaping early FDV intervention and prevention policies. However, the vast number of police recorded FDV events precludes the manual extraction of information with traditional ethnographic/qualitative approaches. One recent research paper commented that “*…there is no systematic way to extract information from these [police] narratives other than by manual review*” (9).

Research within the field of FDV has attempted to understand the relationship between FDV and mental health outcomes for victims such as suicidal ideation, post-traumatic stress disorder and depression. However, there are not many efforts focusing on the prediction of FDV using POI and victim characteristics (e.g., abuse types, mental illness, victim injuries or early signs of abuse, relationship type, drug use) according to a recent meta-analysis of more than 200 FDV studies (10). Furthermore, these studies rely on small to medium sample sizes to draw conclusions, with limited data sources, and often lack actual mental health diagnostic information (10–12). Mental illness can be both a contributor to FDV incidence and increasing evidence suggests that people with mental illnesses are at a greater risk of victimization compared to those without mental illness (7, 13–18).

Given the emergence of predictive policing in recent years (10) and the growth in the availability of “big data,” the possibility of applying sophisticated methods to administrative data collections from law enforcement and health to prevent FDV events has become possible. While text mining has been used to identify crime-related information from online media publications (19–21), few attempts have analyzed police narratives. Recent work has automatically processed police reports to identify information of interest (22–25). In particular, efforts have been made to identify names of offenders, illicit drugs, and weapons from police narratives through named entity extractors with varying degrees of success (F1-scores ranging from 46 to 81%) (22). Other attempts classified police reports as FDV or non-FDV related using an unsupervised clustering method that correctly classified 44% of the reports set aside for manual inspection (24). More recently, deep learning methods have been used to extract mental health related incidents from police narratives with 89% classification accuracy (25).

Predictive analytics have been employed in the crime area with most approaches applying machine learning to Tweets (26), police records (27, 28), or mobile phone behavioral data (29) to forecast local criminal activity or identify crime hot-spots with accuracy ranging from 70 to 81%. A recent attempt to predict time and region-based offenses from police data called DeepCrime (30) used deep neural networks that consistently outperformed standard methods such as Support Vector Regression, Logistic Regression and Multilayer Perceptron with F1-scores ranging from 58.48 to 86.40%.

However, efforts to predict FDV (i.e., whether an individual will hurt a victim within an FDV setting) have returned weak predictive values for repeat victimization and fatal outcomes (31–34). Previous work on the prediction of FDV murder and near-murder over a three-year period was based on 118 police records of deadly FDV cases (31). Each case was classified as standard, medium and high risk through local police risk assessments that involved a series of questions toward the victim resulting in 89% of fatal cases being incorrectly labeled as not high risk (31). A follow up study utilizing the same protocols as Thornton (2017) (31) that attempted to predict 107 cases of FDV murders over a seven-year period, failed to forecast the majority of deadly FDV cases (33). In Australia, efforts to predict repeated FDV in the form of the Domestic Violence Safety Assessment Tool (DVSAT) in New South Wales (NSW) and the Family Violence Risk Assessment Tool (FVRAT) in the Australian Capital Territory (ACT) have also returned weak predictive values (32, 34). FVRAT, a 37-item tool used by the police in ACT when they attend FDV events that weighs their answers to produce a likelihood score of repeated FDV, was not a strong predictor of repeated FDV based on 350 unique cases (32). Similarly, the DVSAT, a 20-item questionnaire consisting of mostly “yes or no” questions administered on victims of FDV which also produces a likelihood score of repeated FDV, was a poor predictor of repeat FDV based on a sample of 24,462 victims highlighting the importance of empirical validation when developing risk assessment tools (34).

A 2019 meta-analysis examining the validity of 39 FDV prediction tools concluded that there is significant room to improve the predictive accuracy for the onset and recurrence of FDV (10) while a retrospective study on prior records of arrestees suggested that mental health markers within police systems can provide information to build more accurate FDV prediction models (35). However, since “*it is notoriously difficult to track FDV trends with police data alone due to under-reporting*” (36), further assessment of the validity of predictive tools could see the incorporation of additional data sources to strengthen their accuracy. An example of such a source is actual mental health diagnoses recorded by health professionals which can be difficult to access due to privacy issues. By linking such data collections with FDV text mined information can allow access to the records of individuals with mental illnesses who have been admitted to a hospital or presented to an Emergency Department with conditions such as schizophrenia. This type of information can be subsequently used as input into predictive models since the police are unlikely to have access to these data and it could potentially strengthen the prediction of FDV.

The lack of adequate predictive tools within FDV highlights the need to develop applications that employ novel methodologies and previously untapped for research data which can equip law enforcement and FDV agencies and other welfare groups with robust knowledge of key FDV factors to develop and apply more effective prevention and intervention initiatives. In this paper, we present a novel methodology that utilizes previously unavailable population level information from police recorded FDV event narratives extracted using text mining and combined with external health data sources which were subsequently input into deep learning methods to predict FDV offense types and to our knowledge, there have not been any previous attempts that follow this approach. We focused on the feasibility of employing deep learning in FDV to predict the risk of related offense types from a unique cross-disciplinary dataset. We show the results of predicting three types of FDV offenses from the application of five deep learning architectures on the combined dataset of text mined information, police fixed fields and linked mental health diagnoses. We also demonstrated how to make these deep learning models interpretable in order to aide in the improvement of the predictive performance by incorporating FDV expert knowledge.

## Materials and Methods

### Data

We obtained 492,393 police recorded FDV events from January 2005 to December 2016 that were flagged in the fixed fields with one of the following tags: “domestic” as the offense type; “domestic violence related” as the associated factor of the police event; “spouse/partner (including ex-spouse/ex-partner),” “boy/girlfriend (including ex-boy/ex-girlfriend),” “parent/guardian (including step/foster),” “child (including step/foster),” “sibling,” “other member of family (including kin),” or “carer” as the relationship status between the victim and the POI. Hereafter, these FDV events covered the following incident categories: assaults, breaches of Apprehended Domestic Violence Orders (ADVO), homicides, malicious damage to property, and offenses against another person (i.e., intimidation, kidnapping, abduction). The FDV events also contained events which the police attended but no particular crime was committed.

Permission to access the FDV events was granted by the NSWPF following ethics approval from the University of NSW Human Research Ethics Committee (HC16558).

#### Text Mined Characteristics From FDV Event Narratives

We previously conducted and published a text mining methodology applied to this FDV dataset that extracted mental illness mentions for POIs and victims, abuse types perpetrated by POIs and victim injuries with F1-scores ranging from 81 to 90% (37, 38). Whilst FDV events can have more than one POI or victim, the current text mining methodology was unable to associate the extracted feature “mention” to a specific POI or victim if more than two individual POIs or victims were present in the same event. Thus, we focused only on those events that included a single POI and a single victim. This resulted in 416,441 FDV events.

We identified 44 different abuse types, 17 different injury types and a total of 126 different mental illnesses for POIs and victims present in 294,024, 145,177, and 64,587 FDV events, respectively, (37, 38) which we combined with the demographic and offending information from the fixed fields in the COPS database for the same cohort at the FDV event level.

#### Linkage to NSW Health Records

We included additional mental health information in the FDV dataset. We obtained ethics approval from the NSW Ministry of Health for the FDV cohort to be linked to two health data collections: the Admitted Patient Data Collection (APDC) which includes records for all hospital separations from all NSW public and private hospitals and day procedure centers, and the Emergency Department Data Collection (EDDC) which provides information on presentations to emergency rooms in public hospitals in NSW. These two collections allowed us to obtain the mental health diagnosis type, age at diagnosis, episode start date, and episode end date. These diagnoses were linked to the FDV cohort by the Center for Health Record Linkage (CHeReL) using probabilistic record linkage. Each individual identified in the linkage was assigned a unique Project Person Number (PPN) to the two external health datasets used for this study.

The diagnoses were linked to the extracted information and the respective fixed fields at the FDV event level by converting the unique Criminal Number Index (CNI, a unique number assigned for anyone who is involved, victim or perpetrator, in an FDV event) to the respective PPN based on a related dictionary provided by CHeReL within a window of one year—before and after the date of the FDV event following consultation with a forensic psychiatrist. This one-year window provides an insight on both currently diagnosed and probable undiagnosed mental states of POIs or victims at the time of the recorded offence by the NSWPF. A description of all the information (text mined characteristics, fixed fields and external mental health diagnoses) used to describe an FDV event can be seen in Supplementary Table 1.

### Offense Definition

Based on a combination of text mined abuse types and recorded incident categories in NSWPF's fixed fields (e.g., “assault,” “homicide”) and following consultation with a forensic psychiatrist, we decided to classify the FDV events under three offense types: “hands-on,” “hands-off,” and “Apprehended Domestic Violence Order (ADVO) breach^1^.” This would enable the classification of the overwhelming majority of the FDV events under any of these offense types delivering large datasets to be used as input into the predictive approach. The definitions of these selected offense types are:

1. “Hands-on offense”: an offense where physical bodily harm was recorded. This can be determined from:
  - The fixed field incident categories (e.g., homicide, assault, actual bodily harm) during the FDV event;
  - The weapon class—a category in which a weapon was used to harm the victim;
  - Certain associated factors—a tag generated by the attending police officer related to the FDV event (e.g., alcohol related); and
  - The text mined physical abuse types (e.g., punching, kicking) and sustained victim injuries (e.g., fracture, cuts).
2. “Hands-off offense”: an offense where other types of non-physical FDV occurred including:
  - Anything classified as non-physical from the fixed fields in the form the malicious damage recorded as an incident type;
  - Text mined non-physical abuse types (e.g., yelling profanities, intimidation via stated threats, stalking).
3. “ADVO breach”: an offense where a POI breached their issued ADVO including:
  - A recorded incident in the fixed fields as “ADVO breaching”;
  - Specific text mined abuse types (e.g., breaching an ADVO, harassment).

Table 1 shows the various features selected to represent the three offense types from the text mined information and the fixed fields.

**Table 1:** The selected features that represent the “hands-on,” “hands-off” and “ADVO breach” offenses from the text mined information and fixed fields.

**Offense type**	**Text mined information**		**Fixed field information**	
Hands-on	Abuse type	Choking, punching, headlocking, assaulting (unspecified), biting, ordering dog attack, dragging, elbowing, grabbing, hair pulling, headbutting, kicking, kneeing, lunging, other (unclassified), physical restraint, sexual assault, pulling, pushing, gagging, scratching, self-harming, shaking, slapping, spitting, stabbing, arm twisting, throwing victim, successful attempt of causing harm with a weapon or object, attempt to use a weapon or object for harm or kill	Generic offense Specific offense	Homicide, assault Common assault, actual bodily harm, manslaughter, grievous bodily harm, murder, shoot_with_no_intent, shoot_with_intent_pre_4_4_94, shoot_with_intent, assault_pre_4_4_94
	Injury type	Bruise, black eye, bleeding, burn marks, cut, fracture, graze, lump, miscellaneous, scratch, stab wound, swelling, tearing nails, broken tooth, soreness	Weapon class Associated factor	Fists, item, speargun, ball, gym equipment, knives, sharp/blunt instrument Bomb explosive related, child abuse related, elder abuse related, firearms related, sexual abuse related
Hands-off	Abuse type	Forced entry into victim's premises, setting fire on premises or objects, chasing, financial control, stalking, ADVO breach, social restriction, blocking exits or pathways for victim, property damage, preventing child access, harassment, intimidation, miscellaneous, possession of victim's personal effects, yelling emotional abuse	Generic offense	Malicious damage
	Threats	Direct threat to damage property, direct threat to harm, direct threat to harm third person, direct threat to kill, direct threat to kill third person, direct threat to self-harm, direct threat to sexual assault, direct threat to steal, veiled threat for sexual assault, veiled threat to harm, veiled threat to harm third party, veiled threat to kill, wish for death	Specific offense	Kidnapping, intimidation, labor exploit, malicious damage, intentional malicious damage, graffiti, negligent act, spike food, bullying/harassment, damaging public fountain, damaging public shrine
ADVO breach	Abuse type	Intimidation, ADVO breach, harassment, forced entry into victim's premises, stalking	Generic offense Specific offense Associated factor	ADVO Breach Intimidation, bullying/harassment Road rage related

*Due to the large number of weapon classes in the fixed fields, we display a few examples. A full presentation of all the weapon classes and the definition of the features can be seen in the Supplementary Table 1*.

**Figure 1:**
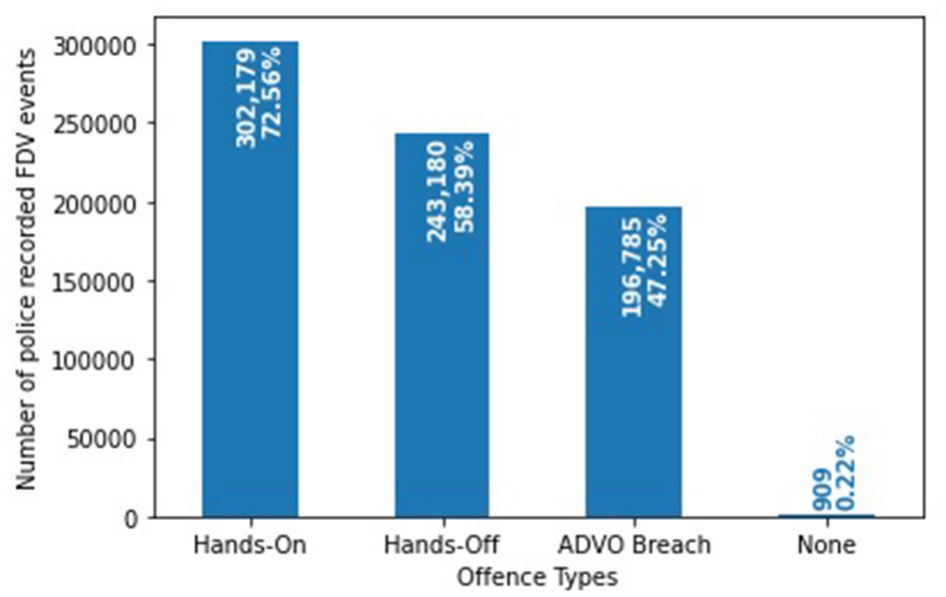
Distribution of the three offense types within the 416,441 police recorded FDV events.

**Figure 2:**
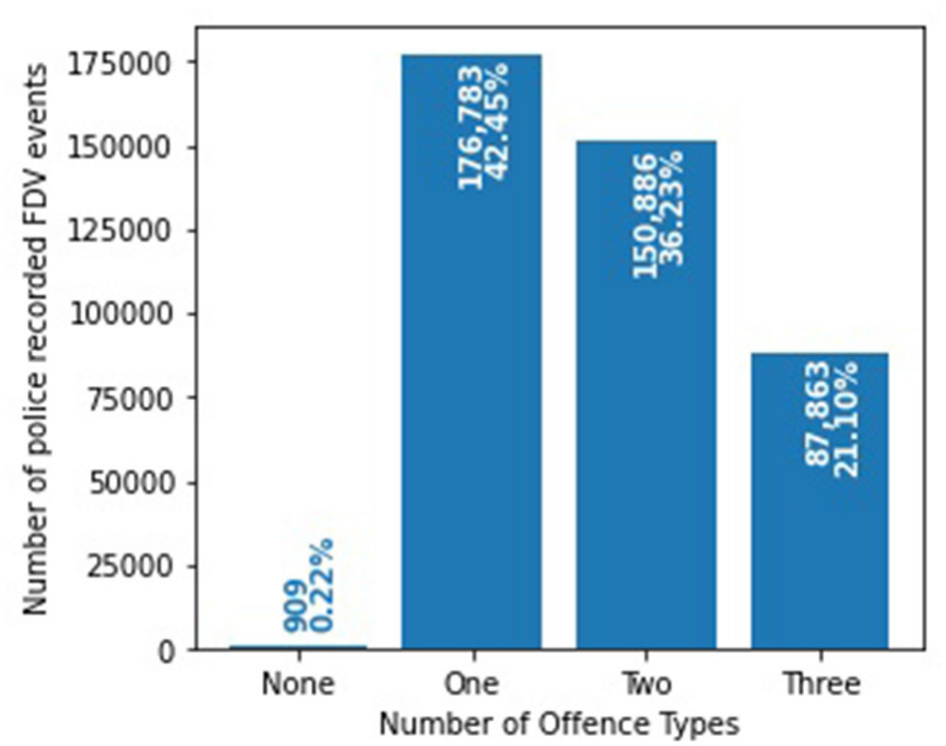
Distribution and intersection of the three offense types within the 416,441 police recorded FDV events.

The FDV events can be classified into more than one of the three offense types (Figure 1). 302,179 (72.56%) FDV events involved a “hands-on” offense while 243,180 (58.39%) are recorded as a “hands-off” offense. A total of 196,785 (47.25%) events involved an “ADVO breach” while 909 (0.22%) did not have any text mined or fixed field information that classified them under any offense type. Figure 2 shows the distribution of the FDV events having one, two or three of the defined offenses. 87,863 (21.10%) FDV events involved all three types of offenses with 150,886 (36.23%) having two offenses and 176,783 (42.45%) involving only one. The dataset included a total of 214,184 unique POIs (i.e., the POI appearing only once in the dataset), and 73,575 (34.35%) were repeat offenders (i.e., appearing in more than one FDV event in the dataset) with a total of 263,084 (63.17%) FDV events. The maximum number of FDV events for a single POI was 40.

### Feature Analysis

**Figure 3:**
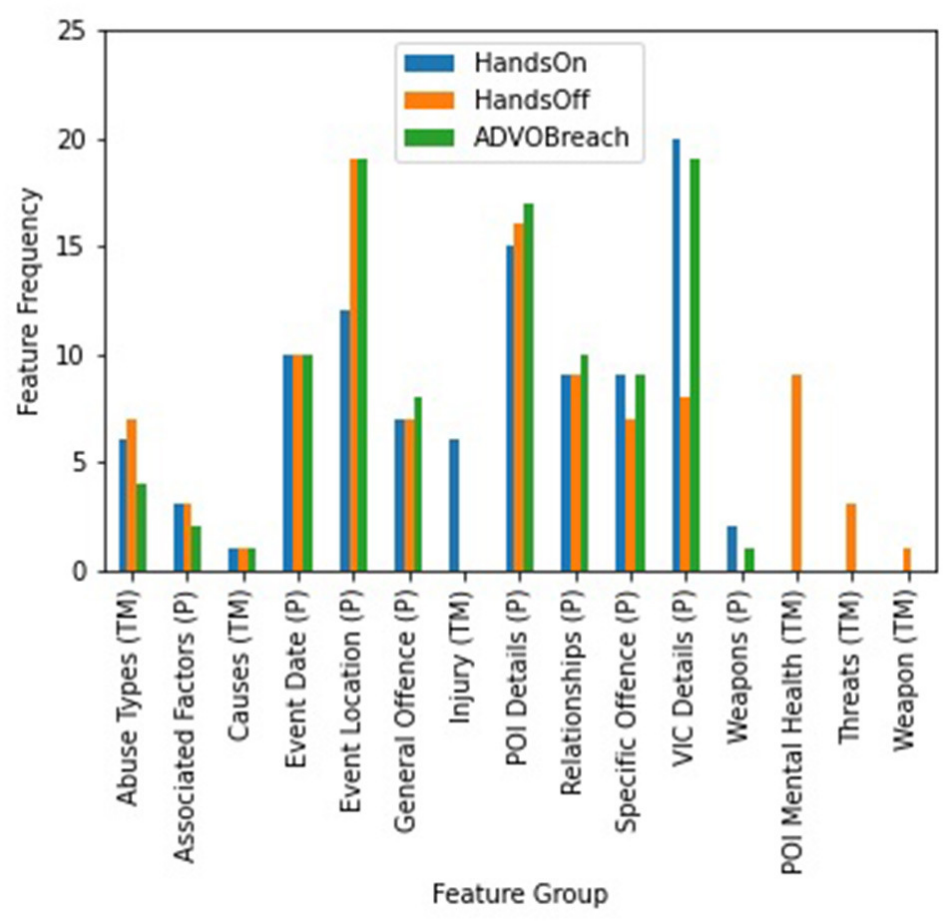
Highest ranking feature categories returned from a chi-square test for the offense types to be predicted from police recorded FDV events. P refers to the fixed field (e.g., premise type, general offense, POI sex) data while TM refers to text mined information (e.g., abuse types, victim injuries).

To measure the significance of the features (text mined information, fixed fields, linked mental health diagnoses) in terms of independence from the defined offense types, the chi-square score was calculated between each feature in the dataset and the output (i.e., offense types). We expanded the data columns (Supplementary Table 1) using binary encoding so that each unique value is one column (e.g., the column abuse type had 44 different abuse types, the column injury type has 17 different injury types, etc.) and then measured how independent each single feature is. For simplicity, we grouped the features into (a) those containing text mined information (e.g., abuse types, mental health mentions); (b) fixed fields subgroups describing the crime aspect of FDV events (e.g., general offense, associated factors); (c) those holding geo-spatio information (e.g., post code, suburb); and (d) those involving POI and victim demographic details (e.g., sex, age). Figure 3 shows 15 out of 18 of these groups containing the top 100 features with the highest chi-square scores for each offense type. The three groups that fall slightly lower in the feature ranking were the APDC and EDDC mental health diagnoses for POIs and victims and the text mined mental illnesses for victims due to their rarity in the dataset (~4%). Fixed field information such as the POI and victim details, general offenses, and relationship status (e.g., “boyfriend/ex-boyfriend”) ranked higher more frequently for “hands-on” and “ADVO breach” offenses while any text mined mention of mental illness for POI including threats and weapons ranked highest for “hands-off” offenses.

### Methodology

#### Data Preparation

**Figure 4:**
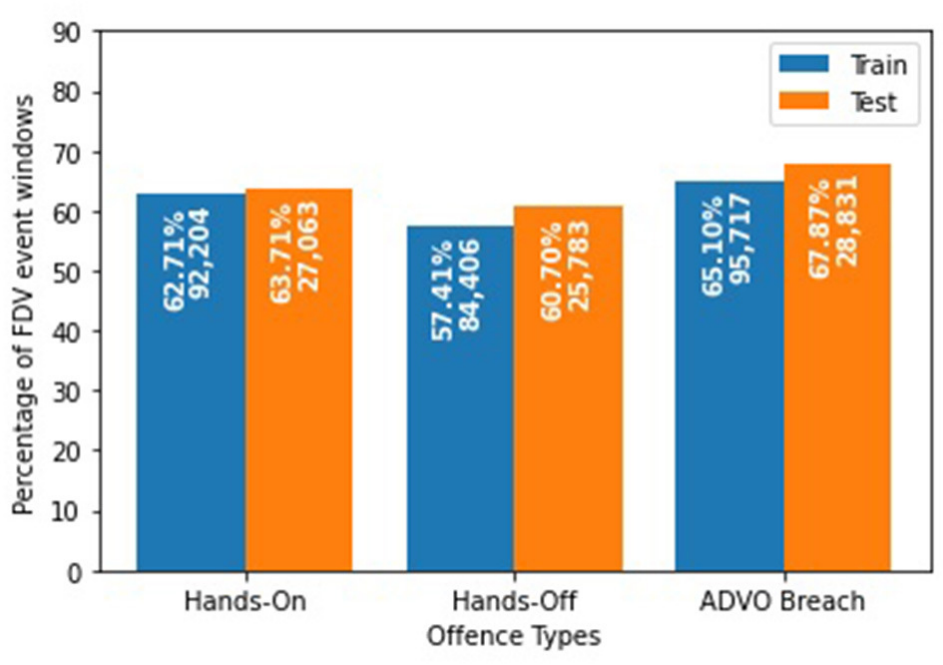
Distribution of the three offense types after data preprocessing in the training and test sets.

To determine the risk of future FDV offense types (“hands-on,” “hands-off,” “ADVO breach”) for individual POIs, we grouped the FDV events by their CNI. We excluded POIs with no CNI (12,748 FDV events) and those POIs involved in only one FDV event (104,609 FDV events). We structured our data as a time series experiment where we predicted the next outcome from a series of previously recorded ones. We created sequential FDV event windows that contained a maximum of three consecutive FDV events involving the same POI prior to the event that we aimed to predict. Despite having details for the event to be predicted, we treat this event as an unseen, future event; thus, only the resulting offense type is selected as the target during the training of our models with no other details being used as feature inputs. The final dataset consisted of 189,509 windows which we split using the FDV event date to create training and testing sets to train and measure the performance of the predictive models. Windows with FDV events for prediction occurring before 2015 were used to train the model (147,030 windows; 77.58%) while those that occurred on and after 2015 were used as the test set (42,479 windows; 22.42%) (Figure 4).

#### Embeddings

The dataset contained multiple multi-valued features (e.g., abuse type, mental health mentions, injuries), high cardinality features (e.g., 3,215 recorded suburbs in the respective fixed field) and sparse features with minimal information (e.g., most weapon classes in the fixed fields had zero values indicating that those weapons were not). Since the application of traditional data encoding techniques (e.g., one-hot encoding, label encoding) does not represent these data well, embeddings were used to transform the data into dense vectors to capture the relationships between features based on learned similarities using neural networks (39). We applied categorical embeddings since they boost the performance and speed of deep learning models when used as input representations of categorical data (39). We transformed the text mined information (e.g., mental illnesses for both victims and POIs), the fixed fields data (e.g., premise type) and the NSW health diagnoses into vector embeddings through transfer learning where information obtained from one task is used in a different one. We trained a deep learning model to map each FDV event to their respective offense type and extracted the learned embeddings to use as inputs for the prediction of future FDV offense types. We used four dense layers with 128, 64, 32, and 16 hidden units, respectively, and trained the model for 30 epochs. The list of feature categories and the embedding size for each category is in Supplementary Table 2.

We also explored the use of word embeddings since the dataset contained descriptive features (e.g., text mined mental health mentions, stated threats). Considering the features as words instead of embedding categories, we “loosely” recreated a narrative for each FDV event aiming to convey the event context better. We used Bidirectional Encoder Representations from Transformers (BERT) (40), a contextual word embedding model that conveys the relationships between words learned from a corpus. BERT was chosen since it distinguishes different meanings of the same word while providing context-dependent results unlike other word embeddings (e.g., Word2Vec, GloVe). We used the publicly available base model^2^ which has 12 layers, 768 hidden units and 12 heads that has been pre-trained on Wikipedia and BookCorpus.

#### Deep Learning Architectures

To predict the risk of a POI committing a future offense type (i.e., “hands-on,” “hands-off,” “ADVO breach”), we structured the experiments as a time series problem where we used a sequence of at most three previous FDV events to predict the outcome of the next committed FDV event. Consequently, for model selection, we focused on variations of Recurrent Neural Networks (RNN), a deep learning architecture that performs well with sequential data. We also include a traditional statistical approach (Naïve Bayes) and a non-sequential base neural network architecture (MLP) to compare with the performance of the sequential models.

As a traditional baseline model, we binary encoded all the features of our FDV dataset and used Gaussian Naïve Bayes, a probabilistic statistical model based on Bayes' theory that functions with the assumption that features follow a normal distribution (41). Along with categorical embeddings, we used the following as deep learning baseline models:

- **Multilayer Perceptron (MLP)**: a rudimentary deep learning architecture that improves upon perceptrons—the primary unit of neural networks (42). We added it to the selection as the most basic deep learning architecture. The base setup used three dense layers with 128, 64, and 32 hidden units, respectively, using ReLu activation (43).
- **Long Short-Term Memory (LSTM)**: a Recurrent Neural Network (RNN) with an internal memory mechanism that works well for processing sequential data (44). LSTM was selected because we utilized sequences of FDV events. A three-layer LSTM architecture was used with 128, 64, and 32 hidden units, respectively.
- **Bidirectional Long Short-Term Memory (Bi-LSTM)**: we also used bidirectional LSTM because bidirectionality allows the model to learn the context of the input data by preserving information from long sequences through simultaneous forward and backward processing. We applied three Bi-LSTM layers with 64, 32, and 16 hidden units, respectively.
- **Bidirectional Gated Recurrent Unit (Bi-GRU)**: a simpler type of RNN that improves on LSTM by implementing a reset and update mechanism making it faster (45). GRU was selected due to its speed when applied to large datasets. We added bidirectionality to improve the model's preservation of context. The base setup consists of three Bi-GRU layers with 64, 32, and 16 hidden units, respectively.

**Figure 5:**
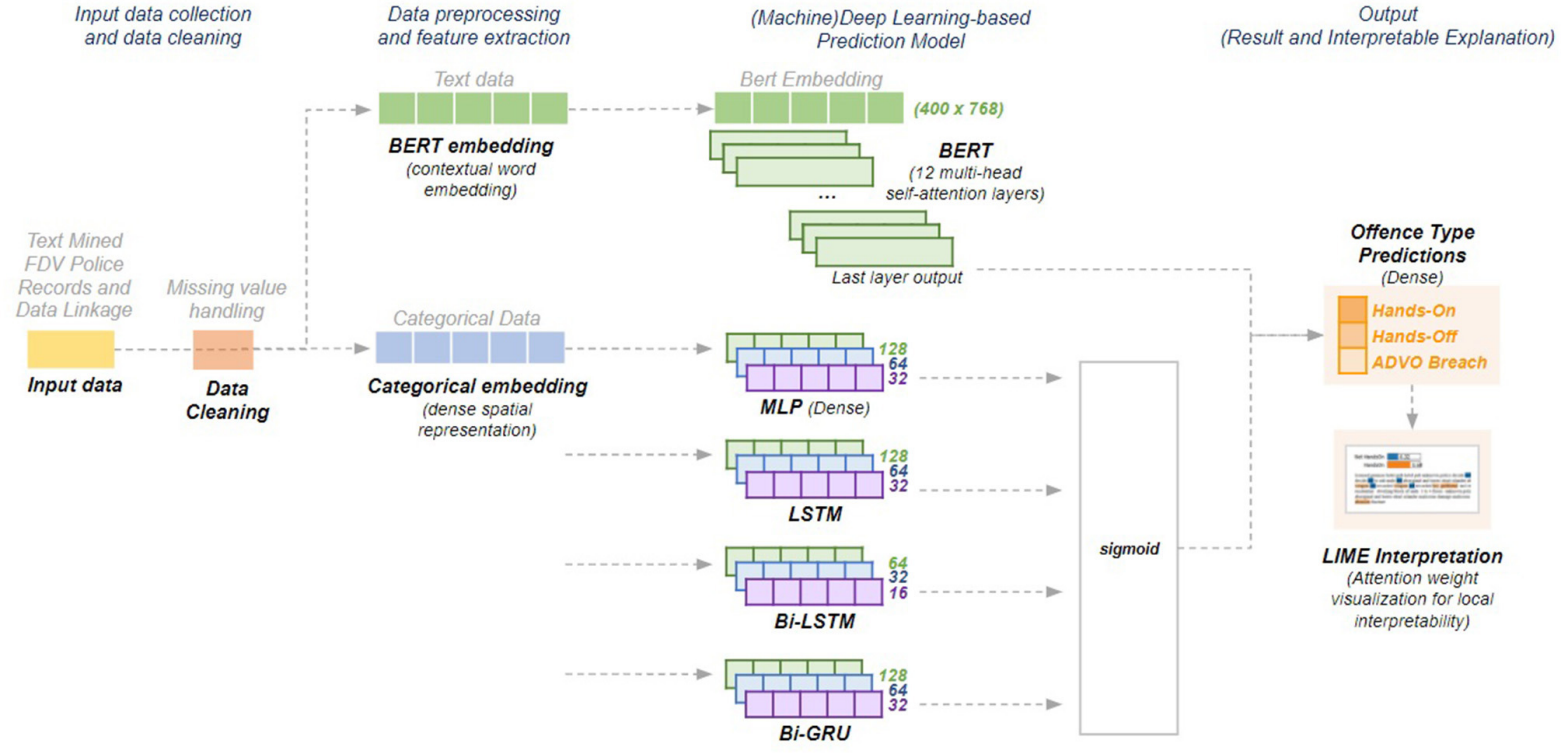
Base schematics for the deep learning models.

Attention-based mechanisms have been gaining popularity to improve on traditional RNNs due to their simplicity and promising results within Natural Language Processing (NLP) tasks. Transformers (46), an attention-only architecture, learn global dependencies between inputs and outputs by drawing attention to important information relative to the task being performed such as areas on an image to concentrate on for visual question-answering, sentences to extract in text summarization, or words to focus on for text classification. Unlike other sequential models, transformers do not process data one at a time. Instead, they use a positional encoding and learn dependencies between the words in these positions using multiple attention layers. Since BERT is a language model that uses transformers, we fine-tuned the pre-trained BERT base model for the predictive task in hand through the addition of a simple classification layer on top of the base model's final attention layer. Due to the extensive time needed to fine-tune BERT (~3 h per epoch), we kept the same hyperparameters throughout the study (maximum length = 400, batch size = 12) and only explored results at 3 and 5 epochs. Figure 5 illustrates the base schematics of the deep learning models used throughout the study.

We included additional model and data setups for our deep learning baseline models to improve the predictive capability of the architectures selected above. We explored a set of varying hyperparameters for our model setups by adding dropouts (50%) and L1 Regularisers (0.01) at various layers to control overfitting issues with the data. Other hyperparameters were kept the same throughout all the models (epochs = 10, batch size = 128). Since the FDV event sequence dataset is large (147,030 windows), we resampled the training data to only 10 or 50% for some models to lessen the possibility of learning noisy or erroneous data which may lead to overfitting. Resampling the data also decreased the training time specially for transformer models that took hours for each epoch. Detailed architecture for each setup variation can be found on Supplementary Table 3.

#### Feature Ablation Study

To assess whether the extracted text mined features and the linked mental health diagnoses from NSW Health contributed to the predictive performance of the deep learning approaches, we implemented an ablation study on the different features added by each data source. We compared results from different setups of MLP and LSTM using three subsets of the FDV event sequences dataset: one containing the fixed field data, another containing both the fixed field and text mined information, and, finally, a third one that contained the fixed field data, the text mined information and the newly added NSW mental health diagnoses. We used the best performing subset for succeeding experiments.

#### Classification Setups

**Figure 6:**
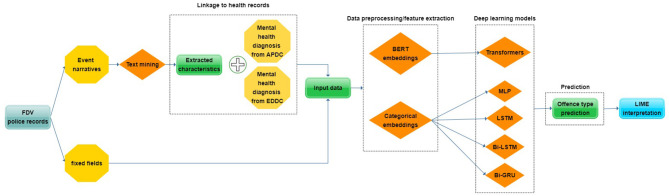
An overview of the methodology used to predict three FDV offense types utilizing a combination of different data sources through a previously used text mining approach, linkage to health records and five deep learning models.

With the aim of predicting the probabilities of three offense types (“hands-on,” “hands-off,” “ADVO breach”) where it is possible that FDV events can fall into more than one offense type, we initially approached the experiments as a *multilabel classification* task. While multilabel classification involves more than one class or category to be predicted, the multilabel approach does not restrict predictions to only one offense type. This method allowed us to save time and resources since one predictive model was able to produce probabilities for all three offense types simultaneously. Nevertheless, multilabel classification provides limited flexibility in fine-tuning models for each individual offense type. To assess the benefit of doing multilabel classification, we broke down the multilabel models into separate *binary classification* models for each of the three offense types. Instead of producing probabilities for “hands-on,” “hands-off,” and “ADVO breach” simultaneously, we turned it into a binary problem where the final probabilities are expanded to “hands-on” and “not hands-on,” “hands-off” and “not hands-off,” and finally “ADVO breach” and “not ADVO breach”. These binary classification models were individually fine-tuned to improve the predictive performance on the specific offense type being predicted. An overview of the entire methodology can be seen in Figure 6.

#### Evaluation Metrics and Result Interpretability

To evaluate the performance of the machine learning approaches, we used the standard definitions of accuracy and Area Under the Curve Receiver Operating Characteristic (AUC ROC) with hamming loss (HL) serving as a measure of accuracy for the multilabel setups and representing the loss of trying to predict the offense types of a future FDV event (i.e., the next FDV event in a window); precision and F1-score were also used for further result interpretability (47) (Supplementary Table 4). We chose to maximize precision over recall (sensitivity) to minimize the profiling of non-offenders.

The performance of the machine learning models was assessed on individual instances through Local Interpretable Model-agnostic Explanations (LIME) (48), an algorithm providing local explanations for individual predictions of any machine learning or deep learning model. In the case of textual data, LIME analyses words that contribute either to or against the generated prediction for a single instance (i.e., FDV event window) (48) and produces insights on the interpretation of the predictive model which can be analyzed further by FDV experts (e.g., forensic psychiatrists, FDV professionals) to assess the qualitative accuracy of the prediction.

## Results

### Feature Ablation Results

**Figure 7:**
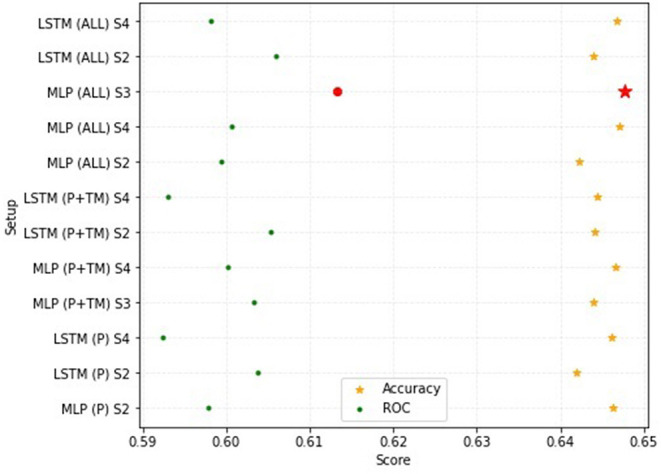
Performance comparison for the ROC and accuracy measures using different subsets of the FDV event sequence dataset; P refers to the fixed field data, TM refers to the text mining data and ALL refers to the fixed field, text mined and NSW Health dataset; S refers to the setup number for each deep learning model.

Using all three data sources (fixed fields, text mined information, actual mental health diagnoses) returned the best overall performance in the multilabel classification ablation study with the best ROC (61.32%) and accuracy (64.77%) (Figure 7). While accuracy scores had minimal differences, ROC scores varied according to the data setup. Using fixed fields (P) generated an ROC range of 59.24 to 60.37% while using both fixed field and text mined information (P + TM) resulted in a slightly wider ROC range (59.30 to 60.54%) (Table 2). In contrast, using all data sources (ALL) increased the ROC range (59.81 to 61.32%.) The lowest ROC (59.24%) and worst accuracy (64.20%) was observed using only the P data with an LSTM setup.

**Table 2:** Feature ablation test results for the two deep learning methods (MLP, LSTM) and their used baseline setups with their corresponding HL, Accuracy and ROC scores for the three subsets of the FDV event sequence dataset: P refers to the fixed field data, TM refers to the text mining data and ALL refers to the fixed field, text mined and NSW Health data (best results are highlighted in green).

**Data**	**Models**	**HL**	**Accuracy**	**ROC**	**Setup description**
P	MLP (Setup 2)	0.3537	0.6463	0.5978	3 Dense; Dropouts
	LSTM (Setup 2)	0.3580	0.6420	0.6037	3 LSTM; Dropouts
	LSTM (Setup 4)	0.3538	0.6462	0.5924	3 LSTM; Dropouts; L1 Reg.
P + TM	MLP (Setup 3)	0.3561	0.6439	0.6033	3 Dense; L1 Reg.
	MLP (Setup 4)	0.3534	0.6466	0.6002	3 Dense; Dropouts; L1 Reg.
	LSTM (Setup 2)	0.3559	0.6441	0.6054	3 LSTM; Dropouts
	LSTM (Setup 4)	0.3555	0.6445	0.5930	3 LSTM; Dropouts; L1 Reg.
ALL	MLP (Setup 2)	0.3577	0.6423	0.5994	3 Dense; Dropouts
	MLP (Setup 4)	0.3530	0.6470	0.6006	3 Dense; Dropouts; L1 Reg.
	MLP (Setup 3)	0.3523	0.6477	0.6132	3 Dense; L1 Reg.
	LSTM (Setup 2)	0.3561	0.6439	0.6059	3 LSTM; Dropouts
	LSTM (Setup 4)	0.3533	0.6467	0.5981	3 LSTM; Dropouts; L1 Reg.

### Multilabel Classification Results

With five or less epochs, the combination of transformer methods and BERT embeddings seemed promising since all the performance metrics (except F1-score) showed huge improvements over the other baseline setups. BERT recorded 35.03% HL, 63.18% ROC, and 68.00% precision. The largest performance increase in ROC scores occurred where transformer models were used, scoring around 63% while the other setups were around 60% (57.27–61.32%) (Table 3). The worst performing deep learning setup was MLP using 10% resampled training data at 39.83% HL, 57.27% ROC score, and 69.70% F1-score while overall, Naïve Bayes had the lowest scores with 55.33% HL, 49.51% ROC, and 43.62% F1-score.

**Table 3:** Detailed results from different setups that used multilabel classification where the deep learning models attempted to learn patterns between the three offense types (best results are highlighted in green).

**Models**	**Resampling**	**Epochs**	**HL**	**ROC**	**F1**	**Precision**	**Setup Description**
Naïve Bayes	-	-	0.5533	0.4951	0.4362	0.6600	-
MLP	-	10	0.3577	0.5994	0.7820	0.6400	3 Dense; Dropouts
	-	10	0.3530	0.6006	0.7751	0.6600	3 Dense; Dropouts; L1 Reg.
	-	10	0.3523	0.6132	0.7696	0.6700	3 Dense; L1 Reg.
	10%	10	0.3983	0.5727	0.6970	0.6702	3 Dense
	10%	10	0.3531	0.5998	0.7758	0.6565	3 Dense; Dropouts
	10%	10	0.3556	0.5985	0.7824	0.6457	3 Dense; Dropouts; L1 Reg.
	50%	10	0.3664	0.5918	0.7485	0.6696	3 Dense layers
	50%	10	0.3532	0.5982	0.7783	0.6537	3 Dense; Dropouts
	50%	10	0.3560	0.6005	0.7703	0.6601	3 Dense; Dropouts; L1 Reg.
LSTM	-	10	0.3561	0.6059	0.7640	0.6700	3 LSTM; Dropouts
	-	10	0.3533	0.5981	0.7739	0.6600	3 LSTM; Dropouts; L1 Reg.
	10%	10	0.3742	0.5818	0.7383	0.6703	3 LSTM
	10%	10	0.3602	0.6026	0.7566	0.6696	3 LSTM; Dropouts
	10%	10	0.3602	0.5849	0.7667	0.6592	3 LSTM; L1 Reg.
	10%	10	0.3542	0.5908	0.7805	0.6497	3 LSTM; Dropouts; L1 Reg.
	50%	10	0.3547	0.6057	0.7636	0.6685	3 LSTM; Dropouts
Bi-GRU	-	10	0.3546	0.6073	0.7640	0.6700	3 Bi-GRU; Dropouts
Bi-LSTM	-	10	0.3574	0.5977	0.7623	0.6700	3 Bi-GRU; Dropouts
	-	10	0.3564	0.5953	0.7683	0.6600	3 Bi-GRU; Dropouts; L1 Reg.
BERT	-	3	0.3503	0.6303	0.7629	0.6800	MaxLen = 400; Batch size = 12
	-	5	0.3524	0.6245	0.7588	0.6800	MaxLen = 400; Batch size = 12
	50%	5	0.3513	0.6318	0.7582	0.6800	MaxLen = 400; Batch size = 12

### Binary Classification Results

In the binary classification of FDV events (e.g., if the POI committed a “hands-on” offense or not) based on the information available from previous FDV events, transformer methods performed comparatively better than the other baseline models for all three offense types in accuracy and ROC scores. The Naïve Bayes baseline consistently returned the worst performance for all three offense types (42.23–46.73% accuracy; 44.44–56.43% ROC; 37.75–50.91% F1-score) while returning the highest precision for “ADVO breach” (81.95%). The results for “hands-on” and “ADVO breach” offenses seemed promising with a 64.56% ROC score, above 65.75% accuracy, and above 75.78% F1-scores. Although BERT records the highest ROC score (64.56%) and second highest accuracy score (65.75%) for the “hands-on” offense type, different fine-tuned MLP setups scored higher for accuracy (65.95%), F1-score (78.03%), and precision (70.00%) (Table 4). LSTM returned the lowest accuracy (62.90%), ROC (61.82%), and F1-score (71.90%) among the deep learning setups.

**Table 4:** Classification performance on predicting “hands-on” FDV offense types (best results are highlighted in green).

**Models**	**Resampling**	**Epochs**	**Accuracy**	**ROC**	**F1**	**Precision**	**Setup Description**
Naïve Bayes	-	-	0.4223	0.4444	0.4294	0.5794	-
MLP	-	10	0.6504	0.6379	0.7455	0.7000	3 Dense; Dropouts
	-	10	0.6595	0.6366	0.7662	0.6800	3 Dense; Dropouts; L1 Reg.
	10%	10	0.6568	0.6356	0.7598	0.6857	3 Dense; Dropouts
	10%	10	0.6487	0.6376	0.7803	0.6486	3 Dense; Dropouts; L1 Reg.
	50%	10	0.6350	0.6291	0.7249	0.6974	3 Dense; L1 Reg.
	50%	10	0.6593	0.6426	0.7662	0.6807	3 Dense; Dropouts; L1 Reg.
LSTM	-	10	0.6551	0.6352	0.7602	0.6800	3 LSTM; Dropouts; L1 Reg.
	10%	10	0.6540	0.6337	0.7655	0.6736	3 LSTM; Dropouts
	50%	10	0.6290	0.6182	0.7190	0.6949	3 LSTM; L1 Reg.
	50%	10	0.6549	0.6298	0.7714	0.6674	3 LSTM; Dropouts; L1 Reg.
Bi-LSTM	-	10	0.6547	0.6329	0.7621	0.6800	3 Bi-LSTM; Dropouts; L1 Reg.
	-	10	0.6504	0.6336	0.7530	0.6900	3 Bi-LSTM, Dropouts, L1 Reg (kernel and bias)
Bi-GRU	-	10	0.6473	0.6319	0.7483	0.6900	3 Bi-GRU; Dropouts
	-	10	0.6565	0.6299	0.7659	0.6800	3 Bi-GRU; Dropouts; L1 Reg.
BERT	-	3	0.6575	0.6456	0.7578	0.6900	MaxLen = 400; Batch size = 12

Predicting whether a POI would commit a “hands-off” offense, BERT worked comparatively well returning the highest accuracy (60.72%) and ROC (57.49%) but with a mid-range precision (62.00%). LSTM still recorded the lowest ROC (53.33%) and F1-score (62.84%) from all the four deep learning approaches with the worst precision returned by MLP (60.75%) (Table 5).

**Table 5:** Classification performance on predicting FDV “hands-off” offense types (best results are highlighted in green).

**Models**	**Resampling**	**Epochs**	**Accuracy**	**ROC**	**F1**	**Precision**	**Setup Description**
Naïve Bayes	-	-	0.4673	0.4708	0.5091	0.5778	-
MLP	-	10	0.6034	0.5560	0.7359	0.6200	3 Dense; Dropout
	-	10	0.5642	0.5575	0.6499	0.6300	3 Dense; L1 Reg.
	50%	10	0.5673	0.5495	0.6665	0.6263	3 Dense; L1 Reg.
	50%	10	0.6067	0.5551	0.7545	0.6075	3 Dense; Dropout; L1 Reg.
LSTM	-	10	0.6036	0.5570	0.7369	0.6200	3 LSTM; Dropouts
	10%	10	0.5928	0.5338	0.7298	0.6110	3 LSTM; Dropouts
	50%	10	0.5459	0.5333	0.6284	0.6244	3 LSTM
	50%	10	0.5939	0.5553	0.7150	0.6229	3 LSTM; Dropouts
	50%	10	0.5892	0.5532	0.7094	0.6217	3 LSTM; L1 Reg.
Bi-LSTM	-	10	0.5952	0.5632	0.7107	0.6300	3 Bi-LSTM; Dropouts; L1 Reg.
Bi-GRU	-	10	0.6027	0.5641	0.7296	0.6200	3 Bi-GRU; Dropouts; L1 Reg.
BERT	-	3	0.6072	0.5749	0.7367	0.6200	MaxLen = 400; Batch size = 12

BERT also returned the highest accuracy (68.82%) and ROC (65.76%) in the prediction of “ADVO breach”. Bi-GRU had the highest F1-score (80.47%) yet the lowest precision (69.00%), a value shared with LSTM and MLP. In addition, MLP had the lowest accuracy (65.88%) and ROC (60.67%) among the deep learning methods (Table 6).

**Table 6:** Classification performance on predicting FDV “ADVO breach” offense types (best results are highlighted in green).

**Models**	**Resampling**	**Epochs**	**Accuracy**	**ROC**	**F1**	**Precision**	**Setup Description**
Naïve Bayes	-	-	0.4504	0.5643	0.3775	0.8159	-
MLP	-	10	0.6826	0.6220	0.8028	0.6900	3 Dense; Dropout; L1 Reg.
	10%	10	0.6588	0.6067	0.7726	0.7054	3 Dense; L1 Reg.
	10%	10	0.6706	0.6195	0.7935	0.6905	3 Dense; Dropout; L1 Reg.
	50%	10	0.6776	0.6121	0.7994	0.6919	3 Dense; Dropout
	50%	10	0.6693	0.6196	0.7827	0.7063	3 Dense; L1 Reg.
LSTM	-	10	0.6824	0.6158	0.8024	0.6900	3 LSTM; Dropouts
	-	10	0.6824	0.6237	0.8039	0.6900	3 LSTM; Dropouts; L1 Reg.
	10%	10	0.6629	0.6277	0.7705	0.7162	3 LSTM; L1 Reg.
	50%	10	0.6742	0.6210	0.7884	0.7048	3 LSTM; Dropouts
	50%	10	0.6768	0.6280	0.7930	0.7014	3 LSTM; Dropouts; L1 Reg.
Bi-LSTM	-	10	0.6836	0.6180	0.8024	0.7000	3 Bi-LSTM; Dropouts; L1 Reg.
Bi-GRU	-	10	0.6774	0.6279	0.7927	0.7000	3 Bi-GRU; Dropouts
	-	10	0.6822	0.6082	0.8047	0.6900	3 Bi-GRU; Dropouts; L1 Reg.
BERT	-	3	0.6882	0.6576	0.8012	0.7100	MaxLen = 400; Batch size = 12

The best performance in multilabel classification was observed in “ADVO breach” which recorded the highest accuracy (69.00%) and F1-score (80.00%) whereas “hands-off” returned the lowest F1-score (73.00%) and the lowest ROC (57.53%) (Table 7). “Hands-on” had the highest ROC (65.43%). In terms of binary classification, “ADVO breach” recorded the highest F1-score (80.12%), ROC (65.76%), and accuracy (68.82%) while “hands-off” returned the lowest F1-score (73.67%), ROC (57.49%), and accuracy (60.72%).

**Table 7:** Performance comparison of multilabel and binary classification setups for each offense type (best results are highlighted in green).

	**Hands-on**			**Hands-off**			**ADVO breach**		
**Setup**	**Accuracy**	**ROC**	**F1**	**Accuracy**	**ROC**	**F1**	**Accuracy**	**ROC**	**F1**
Multilabel (50%)	0.6520	0.6493	0.7400	0.6043	0.5753	0.7300	0.6900	0.6676	0.8000
Multilabel	0.6587	0.6543	0.7600	0.6065	0.5786	0.7300	0.6840	0.6540	0.8000
Binary	0.6575	0.6456	0.7578	0.6072	0.5749	0.7367	0.6882	0.6576	0.8012

From applying either multilabel or binary classification, it was observed that “ADVO breach” had the best F1-score (80.12%) and accuracy (69.00%) from all three offense types whereas “hands-off” seemed more difficult to predict having the lowest ROC (57.49%), F1-score (73.00%), and accuracy (60.43%). Binary classification showed more promise when used on the “hands-off” offense with higher accuracy (60.72%) and F1-score (73.67%) than the multilabel classification setups.

## Discussion

In addition to outright prevention, the holy grail of FDV is to have accurate predictive tools that can be employed to determine the likelihood of future acts of FDV and hence to inform prevention and intervention strategies that aim to protect those exposed to this pernicious behavior. This first effort to link text mining derived information with two administrative data collections represents the start of efforts to combine health, justice, welfare and other data sources that can feed into predictive models to help prevent future FDV events.

We employed several different deep learning approaches to explore whether it was feasible to predict future FDV offenses from police data and whether the predictive performance was increased by incorporating external data sources. The ablation study proved that incorporating features not available in the NSWPF's fixed fields such as text mined information (e.g., abuse types, mental health mentions, victim injuries) from FDV event narratives and linked mental health diagnoses slightly improved the performance of deep learning methods. Such features can convey and describe in detail the severity of an FDV event more than a general list of standardized offenses and weapon classes (i.e., fixed fields). Incorporating NSW Health data further enhanced the predictive results by providing a stronger background on the mental health state of POIs and victims. This offered information on actual diagnoses for severe mental health conditions that might have been missed or unreported when the police attended the FDV event. Although a small increase was observed (~1% ROC), the addition of relevant data from different sources proved to be beneficial for predicting future FDV offense types. Since the aim of this research was to focus on whether it was feasible to employ deep learning to predict future FDV offense types, it should be noted that at this stage no statistical analyses were conducted to document whether this improvement was significant or not.

One potential application of our approach could be the incorporation of mental health data and predictive information into a mobile app used by the police when responding to a FDV event to assess the likelihood of future FDV. Another potential use lies in the profiling of perpetrators or victims that are most likely to inflict or receive abuse, respectively, based on previous data entries which could lead to related FDV and welfare agencies activating those early intervention and prevention initiatives. These public health approaches to FDV could potentially increase the number of saved lives, reduce physical injuries, related health care costs, and poor mental health outcomes for victims and thus improve their quality of life while enabling FDV perpetrators to be directed into respective treatment programs.

### Offense Type Classification

We initially performed multilabel prediction for each FDV window sequence by applying a combination of transformer methods and BERT embeddings with promising results (63.18% ROC; 68.00% precision). BERT was able to learn the context of the data that was “loosely” transformed into a textual narrative better than other deep learning approaches that used categorical embeddings (e.g., MLP, LSTM). Although categorical embeddings were able to find relationships between different feature values (e.g., punch, kick, harassment under abuse types), BERT better captured the non-linear relations between features not captured by other methods that primarily consider these features as discrete, independent embedding components. For example, with categorical embedding, the text mined mental illness mention of “behavioral syndromes associated with physiological disturbances and physical factors” was represented by a single contextualized vector based on its relationship with other values in the mental illness field. BERT, however, further contextualized this mention by representing each word from the given description and relating them to every word from the entire recreated FDV event narrative. Instead of simply considering it as one value among other mental illness mentions, BERT found relationships between parts of the description such as “physical factors” and other fields such as “punching” and “kicking” under abuse types. Consequently, utilizing transformer methods with BERT extracted more value and information from the textual descriptions of text mined features and the NSW Health diagnoses.

Utilizing Naïve Bayes as a traditional baseline model allowed us to compare deep learning methods with a simple statistical probabilistic approach. For both multilabel and binary setups, Naïve Bayes consistently had the lowest scores for all the metrics (42.23–46.78% accuracy; 44.44–56.43% ROC; 37.75–50.91% F1-score) except for precision in “ADVO breach” which had the highest among all setups (81.59%). This indicated that using Gaussian Naïve Bayes with our dataset is worse than chance or random guessing and, despite a high precision in one particular offense type (“ADVO breach”), there is an underlying large number of false negatives among its predictions since the encoded data used as input for the model were very sparse (i.e., most values were zeros) rendering this approach unsuitable in forecasting future FDV offenses.

Despite having the best scores for HL (35.05%), ROC (63.18%), and precision (68.00%), BERT fell short on the F1-score (76.29%) for a multilabel setup. MLP generated the highest F1-score (78.24%), yet achieved a low ROC (59.85%) and precision (64.57%), suggesting that this setup was predicting mostly positives for all the offense types. This can be attributed to the imbalance between positives and negatives for each offense type with all of them having an average of 62.27% positive FDV events classified with such an offense, especially the “ADVO breach” with a range of 65.10 to 67.87%.

Resampling the training data for MLP and LSTM models offered minimal to no improvement, an expected result since these models tend to improve with more data (see Limitations and Future Work). However, resampling for the transformer models returned comparably good results with only 50% of the training data (63.18% resampled ROC score; 63.08% full data ROC score) at half the training time. Consequently, the worst performance in a multilabel setup was observed on MLP using only 10% resampled training data (HL 39.83%, ROC 57.27%, F1-score 69.70%).

Complementing the multilabel classification setup, the binary offense classification returned similarly promising results that could be further explored in future deep learning experiments. Predicting “ADVO breach” was the most promising offense type with 68.82% accuracy and 65.76% ROC score. For “hands-on,” despite transformer methods with BERT having the highest ROC score (64.56%) and second highest accuracy (65.75%), differently fine-tuned MLP setups demonstrated higher scores in other performance metrics (accuracy, F1-score and precision). This could be explained by the difference in the implemented embeddings for both models—word embeddings in BERT and categorical embeddings in MLP. In particular, categorical features (e.g., incident category, further incident category, associated factors, victim's sex, POI's Aboriginal Torres Strait and Islander status) had higher importance for the “hands-on” offense type (Figure 3) therefore, it did not benefit much from the gained context through textual embeddings using BERT. In contrast, the application of BERT for “hands-off” offense types demonstrated a larger improvement over the deep learning baseline models (from 60.27 to 60.72% accuracy and 56.41 to 57.49% ROC).

Since text mined mental health information held a higher rank in importance for this offense (Figure 3), the transformer model could benefit from incorporating both text mined descriptive features from the FDV event narratives and linked mental health diagnoses. “ADVO breach” was the easiest one to predict (68.82% accuracy; 65.76% ROC score) due to the initial FDV event dataset being heavily curated with categorical features that were closely related to such an offense type (e.g., incident category of “ADVO breach,” associated factor of “domestic violence related”). The combination of these features with text mined information that relied heavily on FDV, provided a solid basis of interpretable information that can aid in the prediction of such offense types.

Breaking down the BERT multilabel results for each offense type (Table 7) showed that, “hands-off” was the most difficult to predict with only 60.72% accuracy score, 57.86% ROC score and 73.67% F1-score. Similar features between “hands-off” and “ADVO breach” such as the fixed fields incident categories (“intimidation,” “bullying/harassment”) and text mined abuse types (“intimidation,” “harassment,” “stalking”) made it difficult for the models to distinguish between these two in a multilabel setting. However, for the same reason, the “hands-off” outcome benefited most from being fine-tuned in the binary setup suggesting that perhaps different classification approaches can be used for different offenses. Offenses of similar nature with potentially overlapping sets of features for their definition could benefit more by employing binary setups. However, those that can be highly specific and distinguishable from each other such as predicting only the “hands-on” and “hands-off” offenses simultaneously would perform better in multilabel classification.

### Prediction Interpretation

**Figure 8:**
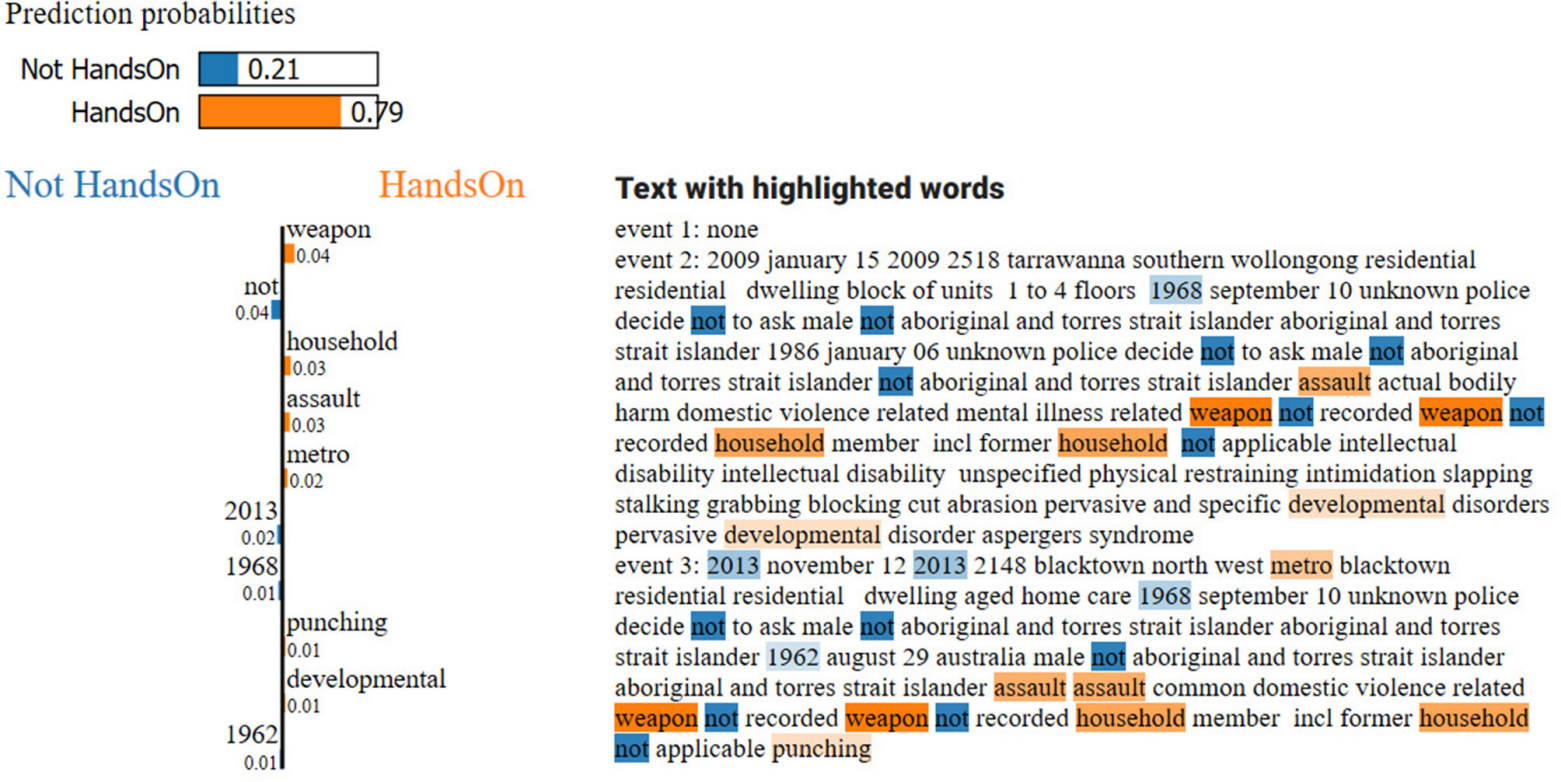
Interpretable explanation from LIME for an instance of predicting a “hands-on” offense type based on two previous FDV events. Top-left are the prediction probabilities generated by the BERT model; a graph displaying the top 10 words that contribute to the negative class “not hands-on” (blue) and the positive class “hands-on” (orange) and their weights of influence is shown on the left; text data with the top 10 words highlighted (darker-colored highlights have larger weights) are on the right.

Interpretability has been one of the top concerns in using deep learning architectures in recent years (47). Despite the generation of state-of-the-art results, most deep learning methods are considered “black boxes” due to the highly complex computation they require. When such methods are used to make predictions that support decision making in fields like medicine or crime, trust in individual predictions becomes crucial (47). For this reason, we utilized LIME to provide a reliable interpretation on offense predictions. For example, an instance with a predicted “hands-on” offense from the BERT setup (Figure 8) made use of two prior FDV events to predict a succeeding “hands-on” outcome for a latter FDV event involving the same POI. BERT classified the instance at 79.00% probability of “hands-on” offense type, verifying that the transformer model worked well for this FDV event sequence. LIME managed to identify several words that influenced the probabilities of each offense type. In the example, “weapon” was one word with a 4.00% weight indicating that the probability generated is not highly dependent on one or two words but rather it was distributed on several ones.

While LIME demonstrated that it can associate words indicating any of the target offenses (e.g., “hands-on” offense has been linked with words such as “weapon,” “assault,” and “punching”), domain experts may have to investigate other words (e.g., “household,” “metro,” and “developmental”—Figure 8) to gain additional insights on the prediction process. Forensic psychiatrists or FDV experts might explore and confirm the possibility that individuals with a particular mental illness (e.g., developmental disorders) could be more prone to either perpetrating or experiencing FDV while law enforcement agencies may question other words that may seem irrelevant to the offenses (e.g., “metro” for “hands-on” offense types) to further assess whether they could offer additional insight. This human expert intervention can be used to process cases where highlighted words do not bear any relevant context to the targeted outcome and removing such cases from the dataset can improve the predictive performance of the model upon re-training. Still, LIME showed tremendous potential in helping to improve predictive tools in the justice health area and it should be considered to be implemented as part of future efforts in that field as a means to gain reliable and interpretable predictive values especially with text-based data such as police and clinical narratives.

### Ethical Considerations

The use of big data and prediction algorithms in the health and justice areas can be controversial. This arises from the public's perception that a “black box” (rather than a human) is making important life-changing decisions such as who gets found guilty, who gets sent to prison and who is released (49), which individuals to target for social security fraud, and which individuals should receive increased police attention (50, 51). Consequently, when developing such predictive applications that rely heavily on private data related to criminal history, it is important to involve the community and other stakeholders to ensure that are acceptable and beneficial to the public and to avoid further stigmatization of groups such as those with mental illness or a specific ethnic group.

The proposed approach here is just the first step in the field of FDV to predict related offenses from population level health and police data. Yet, this type of work has significant implications if it will be integrated and used by law enforcement agencies to assist in crime prevention and intervention, particularly in the sensitive area of FDV. Incorporating the community voice in this work is a necessary step to explore how predictive outcomes can be translated appropriately into the policy and practice of relevant FDV stakeholders and law enforcement agencies. The use of deliberative approaches such as Citizens Juries which involves groups of people (citizens) acting on behalf of the community or Deliberative Forums (52) can provide a process for the public and other stakeholders to inform sensitive areas such as FDV and increase the acceptance of predictive work such as the one described here. It also ensures appropriate oversight and avoids issues such as the possible unfair penalization of vulnerable or over-represented groups and individuals.

### Limitations and Future Work

The proposed approach does not represent the last word in the justice area but a first attempt to develop a pipeline that can be refined and enhanced to improve FDV risk prediction. The study focused more on the feasibility of using deep learning in the field of FDV with a unique dataset rather than attempting to present a clinically usable model which would require extensive testing before usage. Instead, we showed that deep learning can help in identifying risk of future FDV offenses and demonstrated that these scores could be improved by incorporating expert knowledge with the use of deep learning interpretability tools like LIME. Experts could enhance the deep learning architecture based on faults seen on the interpretability tools. Thus, this study can be regarded as a demonstration project of the potential of this approach that needs further refinement. Several limitations are apparent and need to be acknowledged though.

The findings of this study are limited by the way and manner that NSWPF and NSW Health record data. We are not proposing a single solution that can be used for any types of similar data at any region. This is rarely the case in many studies. It is highly likely that regional and local factors could contribute to the modification of such an approach to reflect these variations in both potentially national and international applications of similar nature. In addition, text mined features such as abuse types and injuries seem to be contributing positively to the predictive model performance. However, the text mining methodology might have not been able to capture the extent of abuse expressed with lesser known and infrequent abuse and injury types (e.g., “caging victims,” “malnutrition,” “badly damaged skin”). The extraction approach was based on implemented rules that identified common forms of abuse and their related injuries with narrative text. It is possible that adding rules that aim to identify less common abuse types and injuries, could further increase the performance of the predictive model. Therefore, certain FDV events may lack abusive information that is required to classify them as “hands-on” offense types and could consequently diminish the frequency of “hands-on” offenses within the dataset which could be useful for the model training. Similarly, the “hands-off” offense type included abuse types (e.g., “yelling”) and recorded non-physical incidents (e.g., “harassment/bullying”) in its definition. Hence, it is possible that the text mining methodology and the recorded fixed fields did not contain the more complex cases of non-physical abuse (e.g., socioeconomic forms of violence) which was reflected on the predictive performance for the “hands-off” offense types.

For certain offense types their definitions may overlap which could be affecting the overall performance. In the future, this methodology could be replicated with more specific offenses in mind (e.g., homicide, grievous bodily harm) that sees the incorporation of a targeted set of well-defined features mostly exclusively assigned to each selected offense rather than trying to have three generic offense types.

Although the addition of actual mental health diagnoses proved useful to some degree in the prediction performance, it was linked to a limited number of FDV events. This was to be expected since the attending police officers are exposed to a larger and wider variety of mental illnesses of different severity that do not necessarily require hospitalization as opposed to the more serious diagnoses of individuals that were recorded during hospital or emergency department admissions (e.g., schizophrenia, self-harm).

The incorporation of additional data sources might provide extra information and characteristics for a perpetrator or victim of FDV. A future aim of this work is to link this dataset with additional diverse information from General Practitioner (GP) visits, medication prescriptions, and other justice and welfare data (e.g., child neglect, housing support, prior incarceration history) which can further describe other facets of FDV and has the potential to greatly assist in the labeling of positive and negative FDV events by the current implemented deep learning approaches.

Aside from improvements on the data and on the definition of the predictive outputs, other approaches to machine and deep learning may be also explored. Combining different encoding and embedding techniques may yield substantially different results compared to exclusively using only one embedding approach for each deep learning model. For instance, combining both categorical and BERT embeddings with transformer models may improve the predictive results as different types of information would be captured by both approaches. Furthermore, direct use of the original FDV event narratives would benefit the BERT transformer models greatly as opposed to the current loosely recreated narrative but, for confidentiality purposes, the original FDV dataset was not accessible anymore. A better structure of the recreated narrative instead of simply concatenating all features might be a preferable solution for the future. Exploring other machine learning models such as random forests or graph neural networks or a combination of different methods may also prove beneficial specially with different information coming from different data sources. Other interpretability tools (e.g., SHAP—SHapley Additive exPlanations) (53) could be explored in conjunction with LIME to improve the predictive results of deep learning models through incorporating feedback from FDV and other related experts in this area.

With a unique cross-disciplinary dataset, various frames of questioning could be explored as well. Future studies may focus on the identification of the type of abuse for repeat victims involved in more than one FDV event or utilize only FDV events where POIs and victims have mental health information (text mined and obtained from external health sources) to make mental health well-being in the justice health perspective the focal point. Risk of reoffending or repeat victimization based on similarity of perpetrator or victim profiles from previously recorded FDV events could also be an interesting study.

## Conclusion

FDV is a global problem with significant social, economic and health consequences for victims. Despite the severity of this complex phenomenon, there is an absence of effective tools that can predict the probability of future FDV based on POI and victim features. We examined the feasibility of utilizing deep learning in the area of FDV on a population level dataset of half a million police recorded events to predict offense types. This study applied five different deep learning models on text mined and standardized information from police FDV events and actual mental health diagnoses from emergency and hospital admissions to predict “hands-on,” “hands-off,” and “ADVO breach” offense types. The best outcome performance was returned by the transformer model with BERT embeddings (69.00% accuracy, 66.76% ROC) for “ADVO breach” in a multilabel classification setup. Overlapping features for two offense types (“ADVO breach,” “hands-off”) affected the performance of the methodology and there is a need to incorporate additional FDV related information (e.g., GP visits, housing support) that can contribute to better predictive value. Furthermore, incorporating expert knowledge through the evaluation of interpretability tools could further boost predictive performance. Our results indicate that such a cross-disciplinary pipeline in the justice health area involving a fully evaluated text mining methodology, data linkage and deep learning modeling could assist in the prediction of FDV offenses and in the development of nuanced prevention and intervention activities by law enforcement and FDV agencies.

## Data Availability Statement

The data analyzed in this study is subject to the following licenses/restrictions: the datasets used for this work are not publicly available due to the inclusion of sensitive information and data confidentiality issues. Requests to access these datasets should be directed to George Karystianis, g.karystianis@unsw.edu.au

## Ethics Statement

Permission to access the police recorded DV events was granted by the NSWPF following ethics approval from the University of NSW Human Research Ethics Committee (HC16558).

## Author Contributions

GK: study conception and initialization, literature review, data collection, data linkage, result interpretation, manuscript preparation, and revision. RC: study design, data linkage, data preparation, data analysis, result interpretation, manuscript preparation, and revision. SH: study design and supervision, data analysis, result interpretation, and manuscript revision. JP: study design, data analysis, and manuscript revision. TB: study conception, initialization, and manuscript revision. All authors contributed to the article and approved the submitted version.

## Conflict of Interest

The authors declare that the research was conducted in the absence of any commercial or financial relationships that could be construed as a potential conflict of interest.

## Footnotes

^1^An ADVO is issued by the court and prohibits physical and non-physical contact between a POI and a victim.

^2^ https://tfhub.dev/tensorflow/bert_en_uncased_L-12_H-768_A-12/1

## Abbreviations

ACT: Australian Capital Territory
ADVO: Apprehended Domestic Violence Order
APDC: Admitted Patient Data Collection
ATSI: Aboriginal Torres Strait Islander status
AUC: Area Under the Curve
BERT: Bidirectional Encoder Representations from Transformers
Bi-GRU: Bidirectional Gated Recurrent Unit
Bi-LSTM: Bidirectional Long-Short Term Memory
CHeReL: Centre for Health Record Linkage
COPS: Computerized Operational Policing System
CNI: Criminal Number Index
DVSAT: Domestic Violence Safety Assessment Tool
EDDC: Emergency Department Data Collection
FDV: Family and Domestic Violence
FVRAT: Family Violence Risk Assessment Tool
GP: General Practitioner
HL: Hamming Loss
LIME: Local Interpretable Model-agnostic Explanations
LSTM: Long-Short Term Memory
MLP: Multilayer Perceptron
NSW: New South Wales
NSWPF: New South Wales Police Force
POI: Person of Interest
PPN: Personal Project Number
RNN: Recurrent Neural Network
ROC: Receiver Operating Unit
SHAP: SHapley Additive exPlantations.
